# A prognostic model for ovarian neoplasms established by an integrated analysis of 1580 transcriptomic profiles

**DOI:** 10.1038/s41598-023-45410-x

**Published:** 2023-11-08

**Authors:** Yanjiao Hua, Du Cai, Cole Andrea Shirley, Sien Mo, Ruyun Chen, Feng Gao, Fangying Chen

**Affiliations:** 1 The Reproductive Hospital of Guangxi Zhuang Autonomous Region, Nanning, 530021 China; 2 Department of Colorectal Surgery, The Sixth Affiliated Hospital of Sun Yat-Sen University, Guangzhou, 510655 China; 3 Guangdong Institute of Gastroenterology, Guangzhou, 510655 Guangdong Province China; 4 Guangdong Provincial Key Laboratory of Colorectal and Pelvic Floor Diseases, The Sixth Affiliated Hospital, Sun Yat-Sen University, Guangzhou, 510655 Guangdong Province China; 5 Sun Yat-Sen University, Guangzhou, 510080 Guangdong Province People’s Republic of China; 6 Department of Obstetrics and Gynecology, The First Affiliated Hospital, Sun Yat-Sen University, Guangzhou, 510080 Guangdong Province People’s Republic of China

**Keywords:** Cancer, Oncology

## Abstract

Even after debulking surgery combined with chemotherapy or new adjuvant chemotherapy paired with internal surgery, the average year of disease free survival in advanced ovarian cancer was approximately 1.7 years^1^. The development of a molecular predictor of early recurrence would allow for the identification of ovarian cancer (OC) patients with high risk of relapse. The Ovarian Cancer Disease Free Survival Predictor (ODFSP), a predictive model constructed from a special set of 1580 OC tumors in which gene expression was assessed using both microarray and sequencing platforms, was created by our team. To construct gene expression barcodes that were resistant to biases caused by disparate profiling platforms and batch effects, we employed a meta-analysis methodology that was based on the binary gene pair technique. We demonstrate that ODFSP is a reliable single-sample predictor of early recurrence (1 year or less) using the largest pool of OC transcriptome data sets available to date. The ODFSP model showed significantly high prognostic value for binary recurrence prediction unaffected by clinicopathologic factors, with a meta-estimate of the area under the receiver operating curve of 0.64 (*P*  =  4.6E-05) and a D-index (robust hazard ratio) of 1.67 (*P*  =  9.2E-06), respectively. GO analysis of ODFSP’s 2040 gene pairs (collapsed to 886 distinct genes) revealed the involvement in small molecular catabolic process, sulfur compound metabolic process, organic acid catabolic process, sulfur compound biosynthetic process, glycosaminoglycan metabolic process and aminometabolic process. Kyoto encyclopedia of genes and genomes pathway analysis of ODFSP’s signature genes identified prominent pathways that included cAMP signaling pathway and FoxO signaling pathway. By identifying individuals who might benefit from a more aggressive treatment plan or enrolment in a clinical trial but who will not benefit from standard surgery or chemotherapy, ODFSP could help with treatment decisions.

## Introduction

Ovarian cancer (OC) has the fourth highest morbidity and the third highest death among gynecological neoplasms globally. Despite the fact that the prevalence of OC has been declining, it still has the second highest death rate, which is on the rise. The mean year of disease free survival (DFS) in advanced ovarian cancer was about 1.7 years^1^, even after debulking surgery paired with chemotherapy or novel adjuvant chemotherapy along with internal surgery. After the current standard treatment, approximately 70% of patients achieve complete remission (CR); nevertheless, 30–40% of these patients relapse within 12 months^2,3^. Moreover, it is standard practice to administer adjuvant chemotherapy for 6–8 cycles following surgery in patients with ovarian cancer^4^. This treatment regimen typically spans a duration of around six months. If the tumor relapses within 6 months after completing the last cycle of chemotherapy, it is classified as platinum-resistant, indicating a lack of response to platinum-based chemotherapy. The identification of nonresponders and patients with primary platinum resistance (recurrence within 6 months after the last chemotherapy cycle) is crucial for improving the overall survival of individuals with serous ovarian carcinoma. It also has implications for subsequent treatment plans, including the choice of alternative chemotherapy agents and targeted therapies^5^.

Following the widespread application of high-throughput next-generation molecular profiling methods, multiple researches have uploaded transcriptome profiles of OC to the public medical database^6^. These gene expression profiles were evaluated using a variety of statistical methods in order to identify differentially expressed genes (DEGs) in various subgroups and create predictive models or progression stage classifiers based on DEG expression levels^7–12^. While the biological relevance of these reported classification systems is supported by overlap between these models, their prognostic utility has not been optimized.

Previous studies ^13–15^ established predictive biomarkers and prognostic models using numerous heterogeneous independent datasets, most with small samples and without appropriate validation. A meta-analysis of data sets from several cohorts was used to construct a predictive model for chemotherapy resistance^16^. Its therapeutic use was limited by the inherent bias resulting from variability between included research and the vast number of selected genes. In addition, the prognostic model lacked comparisons to established clinicopathological factors and did not account for clinical pathological features.

To address these difficulties, we developed the Ovarian Cancer Disease Free Survival Predictor (ODFSP) model using the top-scoring pair (TSP) algorithm. TSP algorithm classifies phenotypes according to the relative expression of a pair of genes^17^. Thus, it is resistant to potential batch effects, profiling platforms, and normalization approaches and has been tested as the feature selection method for various machine learning models^18^. The ODFSP model employed a unique sample of 159 ovarian serous neoplasms that were profiled using both microarray and sequencing methods. Using an independent set of ovarian transcriptome profiles from 1580 primary resected patients, we show that ODFSP is a reliable single-sample predictor of early recurrence—1 year or less—after surgery. This could be used as a tool to aid doctors in their decision-making.

## Materials and procedures

**Figure 1:**
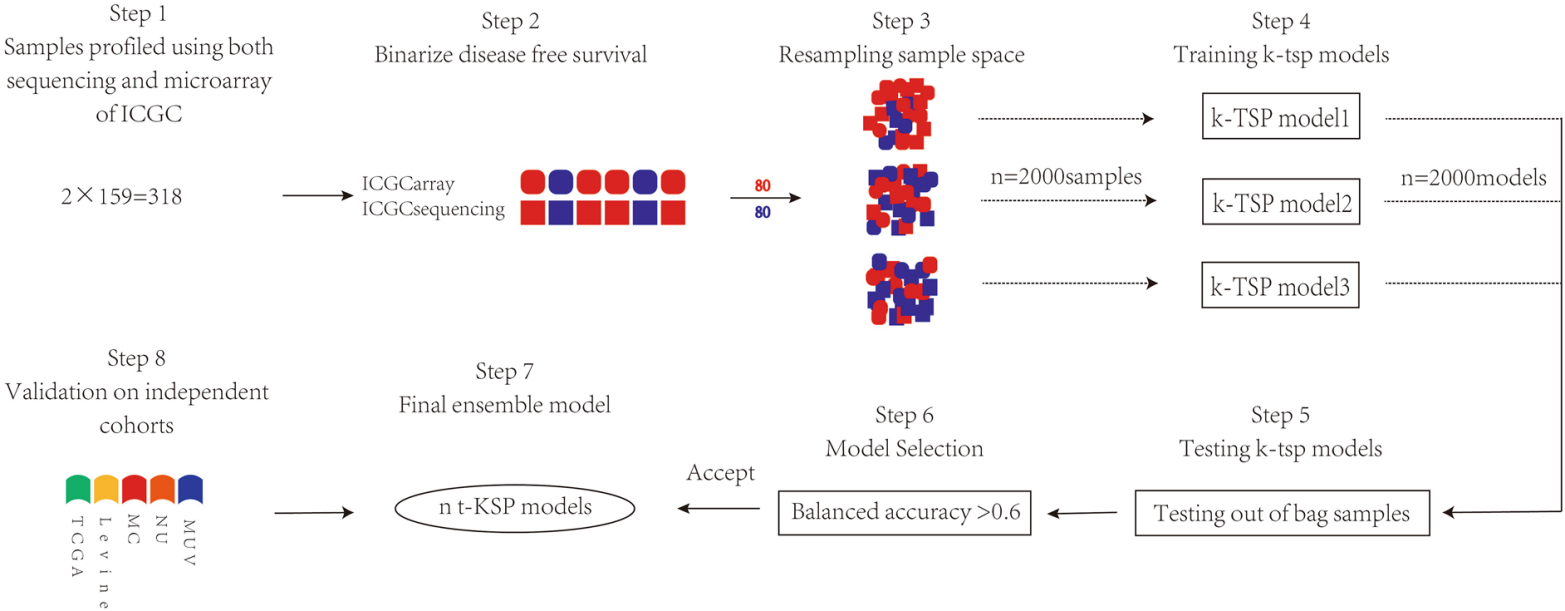
Pipeline demonstrating the development of the Ovarian Cancer Disease Free Survival Predictor (ODFSP).

The meta-analytical pathway for developing the ODFSP model and assessing its predictive usefulness is shown in Fig. 1.

### Collection of datasets

**Figure 2:**
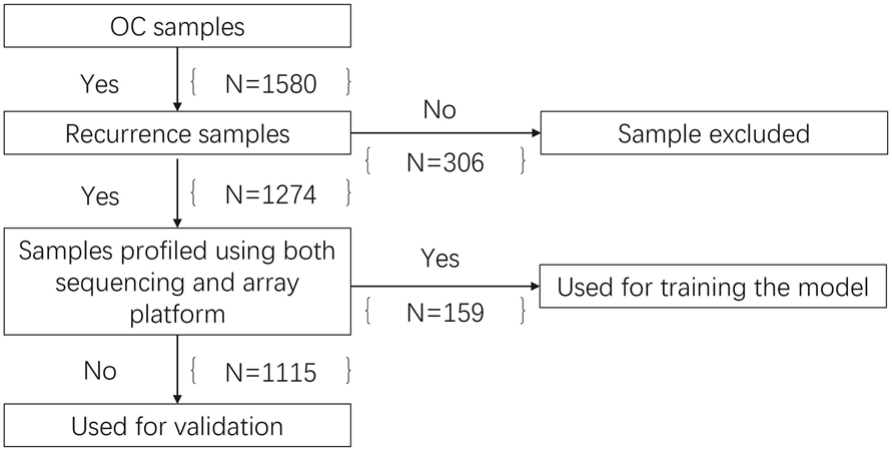
Flowchart outlining the requirement for enrolling samples for ovarian cancer (OC) samples. Six data sets were combined to create a total of 1580 OC samples. After samples were dichotomized into groups with a high and low risk of recurrence, they were filtered based on the availability of DFS and clinical information. The filtering criteria were met by a total of 1274 samples, of which 159 samples were utilized for training and 1115 samples for validation.

We reviewed the literature and selected 6 data sets from the public domain that contained 1580 patients with ovarian cancer and transcriptome data for ovarian cancer (Data Supplement S1). We selected samples using the criteria of the availability of disease free survival (DFS) and no loss of follow-up within one year (Fig. 2), prior to dichotomizing patients into high- and low-recurrence groups on the basis of a DFS threshold of 1 year. The various cohorts presented with similar clinical symptoms and had interventions of curative surgery followed by adjuvant chemotherapy (Data Supplement S1 and S2).

### Establishment of predictive model

We used gene expression patterns from 159 samples from patients with ovarian carcinoma of the International Cancer Genome Consortium (ICGC) cohorts to build a robust predictor for early recurrence. In the ICGC cohorts, both microarray and sequencing technologies were employed to tumors. As previously reported^8,9,19–21^, human research ethics permission was given. Approximately 50 percent of the patients in the training cohort eligible for surgery relapsed within a year, thus a one-year threshold was set to distinguish which patients with ovarian neoplasms had a high risk of recurrence.

We converted the original gene expression profiles into binary gene pair barcodes to make the training and validation sets' gene expression profiles comparable. In the SwitchBox package (version 1.2.0), we implemented the k-Top score disjoint pairs classifier predictor utilizing the Wilcoxon rank sum technique as a filtering function^22^. Two permutation tests were used to see if the ODFSP model’s predictive value could be attained just by chance (Appendix A1 and A2).

### Comparison of the performance of the ODFSP

For examination and statistical comparison of the ODFSP performance, meta-analysis was done on the sequencing cohorts, array-based cohorts, and overall combined cohorts of OC. Depending on the outcome variable—recurrence within a year or less—patient samples were divided into two groups. Samples of lost to follow-up within a year were excluded from meta-estimate of the area under the receiver operating characteristics curve (AUROC). The AUROC plots sensitivity versus 1-Specificity and is used as a metric for the assessment of the model's discriminatory ability^23^. The AUROC curve estimate was calculated using the pROC package (version 1.18. 0)^24^, and the *P* value was calculated applying the Mann–Whitney test statistics to determine whether the AUROC curve estimate is significantly different from 0.5 (random classifier). The random effect model^25^ implemented in the survcomp package (version 1.42.0)^26^ was used to calculate the AUROC meta-estimate.

### The measure of heterogeneity of various cohorts

The dissimilarity index (D-Index), a robust estimate of the classic Cox hazard ratio, was used to estimate the prognostic value and statistical significance of the survival difference between the predicted groups. The fundamental advantage of D-index over hazard ratio (HR) is that it provides a scale-free measure of separation between two independent survival distributions that can be interpreted under the proportional hazards assumption^27^. In a meta-analysis situation where it is imperative to account for the heterogeneity of multiple cohorts, the D-index is a good approximation of prognostic value and a high D-index reflects a strong separation between the survival distributions of different groups. We also utilized the concordance index (C-index), which measured the ability of the predictor to order the events by estimating the fraction of correctly ordered pairs out of all comparable pairs in the dataset^28^.The survcomp package was used to calculate the D-index and C-index. Using the random effect model implemented in the survcomp package, we estimated the meta estimate of the D-index and C-index for the OC sequencing cohorts, the OC array-based cohorts, and the combined ovarian serous cancer sequencing and array-based cohorts. The median DFS score was used to divide patients into low- and high-risk groups. The survminer package (version 0.4.9)^29^ in R was used to plot Kaplan–Meier curves, and the *P* values from the log-rank test were reported.

### Early recurrence prediction model based on clinicopathologic features

We developed the clinical model by fitting a logistic regression model to age and FIGO stage data from ICGC sequencing, ICGC array, The Cancer Genome Atlas (TCGA), Levine cohort, Mayo Clinic cohort, Niigata University, and Medical University of Vienna cohorts.

### Other classifiers

Although some studies published multiple biomarkers for the recurrence of ovarian cancer, there were no prediction models for DFS of OC. Hu et al. used the LASSO Cox regression model in the TCGA database to create a multi-gene signature for 1-, 3-, and 5-year overall survival. We used the reported coefficients of the 8 classifier genes as weight factors in the sig.score function in the genefu R package (version 2.24.2) to generate the Hu signature scores^30^. Also, using the scikit-learn package in Python 3.7, machine learning approaches such as the logistic regression algorithm were evaluated in constructing a DFS prediction model. We used five validation cohorts to calculate the C-index and D-index for the three classifiers, ignoring the cohorts used for training by ODFSP and other classifiers in comparison. Furthermore, we used the survcomp package (version 2.13.0) to construct ODFSP for comparison of meta-estimates between each classifier's C-index at *P* < 0.05 (one-sided t test).

### Analysis of gene set enrichment

GO enrichment and KEGG analyses were performed using the ClusterProfiler package (version 4.2.2) in R^31^. RunGSAhyper function in the piano package (version 2.8.0) was used to perform gene set enrichment analysis to categorize genes in ODFSP^32^.

## Results

### Predictive model for DFS

**Figure 3:**
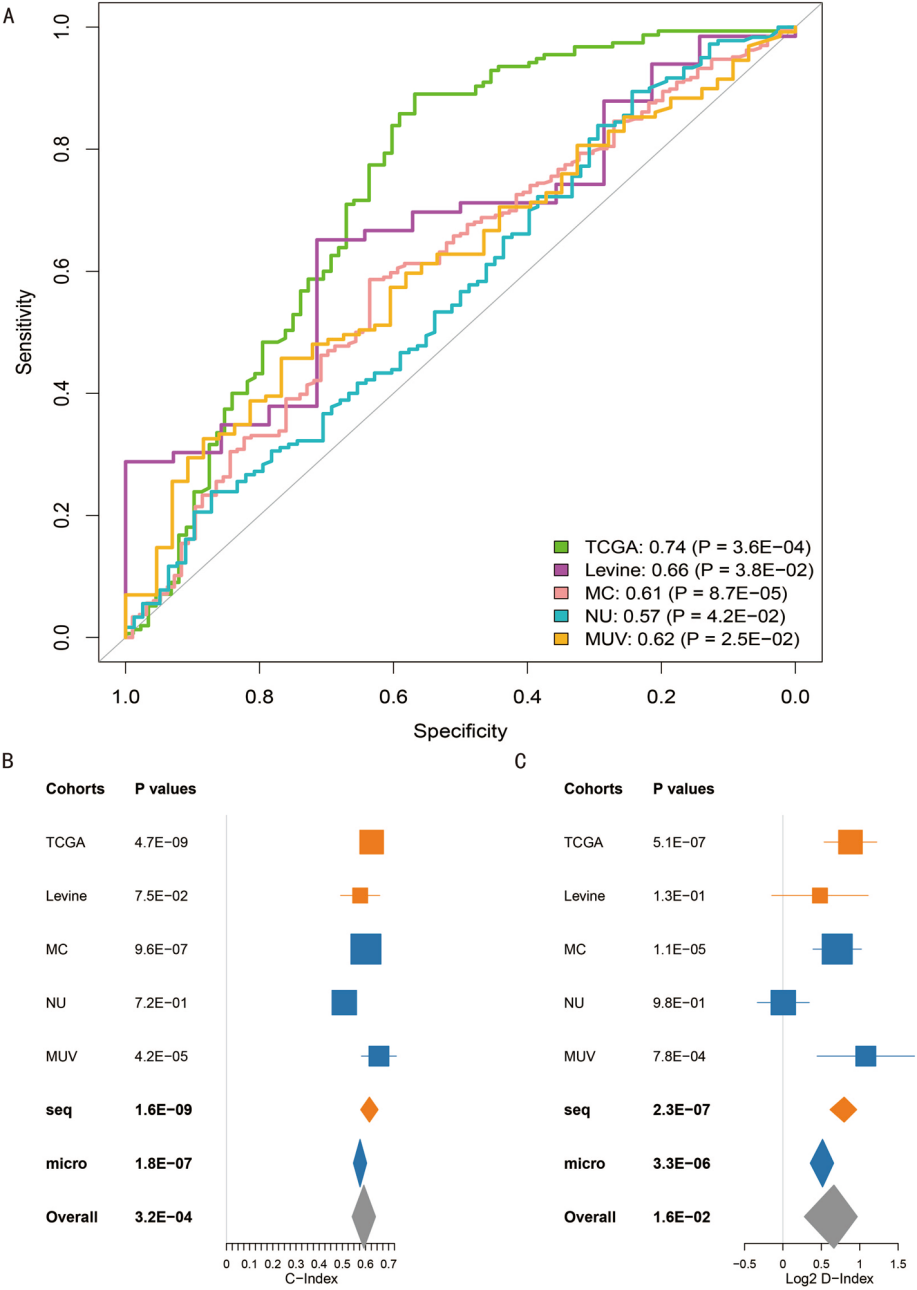
Predictive value of the Ovarian Cancer Disease Free Survival Predictor (ODFSP) for early recurrence.

159 microarray and sequencing transcriptome profiled ICGC cohort samples were adopted to train the ODFSP model to identify patients who relapse within a year. We looked at the ODFSP score’s predictive significance in two different sequencing cohorts: the TCGA Ovarian Cancer and Levine cohorts, as well as three independent array-based cohorts: Mayo Clinic (MC), Niigata University (NU), and Medical University of Vienna (MUV). We initially calculated the AUROC for each data set separately to see if early recurrence had any predictability. The ROC curve was shown in Fig. 3A and the exact count of samples predicted in ODFSP in each cohort was displayed in Data Supplement S3. ODFSP was significant overall (AUROC, 0.64, *P* = 4.6E-05), but it was higher in data sets generated using sequencing platforms than in data sets generated using microarrays (AUROC, 0.72 *v* 0.60 for sequencing and array data sets, respectively), proposing that RNA sequencing may be a more suited assay for ODFSP than microarray platforms. In all cohorts, ODFSP was substantially associated with early recurrence (AUROC ∈ [0.57, 0.74], *P* < . 05; Fig. 3A).

To determine if the ODFSP model’s early relapse predictive value can be attained fortuitously, we first produced AUROC meta-estimates by shuffling the class labels—early recurrence—1000 times and using the same training technique as the ODFSP model. Lower balanced accuracy (BAC) of random models showed not a single random model was capable of producing a prediction value greater than or equal to ODFSP (*P* < 0.001; Appendix A1a), indicating that gene expression profiles were strongly linked with recurrence. Upon random allocation of genes to the ODFSP, we tested whether the gene pairings nominated in the ODFSP model were robustly related with early recurrence events. It was discovered that the genes selected in ODFSP had significantly more predictive efficacy than random gene models (*P* < 0.001; Appendix A1b), indicating that the ODFSP gene collection is biologically relevant.

### The ODFSP model’s prognostic value

As measures for the degree of separation for models of survival data, C-index and D-index were generated using DFS data for all cohorts to assess the ODFSP model’s predictive efficacy. Overall, the C-index is significant (0.58; *P* = 3.2E-04; Fig. 3B), probably reaching 0.60 (*P* = 2.1E-17) if the clear deviating result in NU cohort was omitted. The ODFSP prognostic value was elevated for sequencing data sets compared to arrays (C-index, 0.61; *P* = 3.7E-10 *v* C-index, 0.57; *P* = 1.0E-06, respectively; Fig. 3B), which was consistent with early recurrence prediction results. ODFSP D-index was strong and significant overall (D-index = 1.50; *P* = 2.2E02; if NU removed, D-index = 1.67; *P* = 9.2E-06, Fig. 3C) and stronger for sequencing data sets as compared to arrays (D-index, 1.77 *v* 1.42, Fig. 3C).

**Figure 4:**
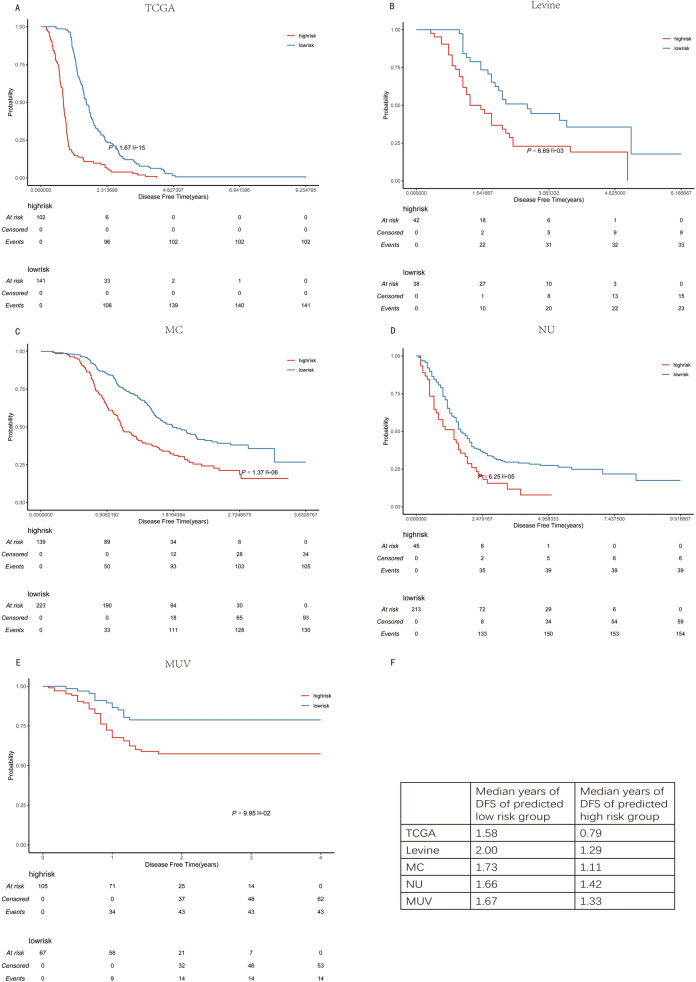
Kaplan Meier overall survival curves for (**A**) The Cancer Genome Atlas (TCGA), (**B**) Levine cohort, (**C**) Mayo Clinic cohort (MC), (**D**) Niigata University (NU), and (**E**) Medical University of Vienna cohorts (MUV) show the *P* values from log-rank test. (**F**) Disease free survival of predicted low- and high-risk groups aare shown in the table.

With division of patients into low and high-risk groups of early recurrence, Kaplan–Meier curves were plotted for each cohort to illustrate the prognostic value of ODFSP (Fig. 4A–E). In all sequencing cohorts and microarray cohorts (*P* < 0.05), DFS was significantly different between risk groups with a 7-month difference in median DFS (Fig. 4F).

### DFS prediction using a clinicopathologic model

**Figure 5:**
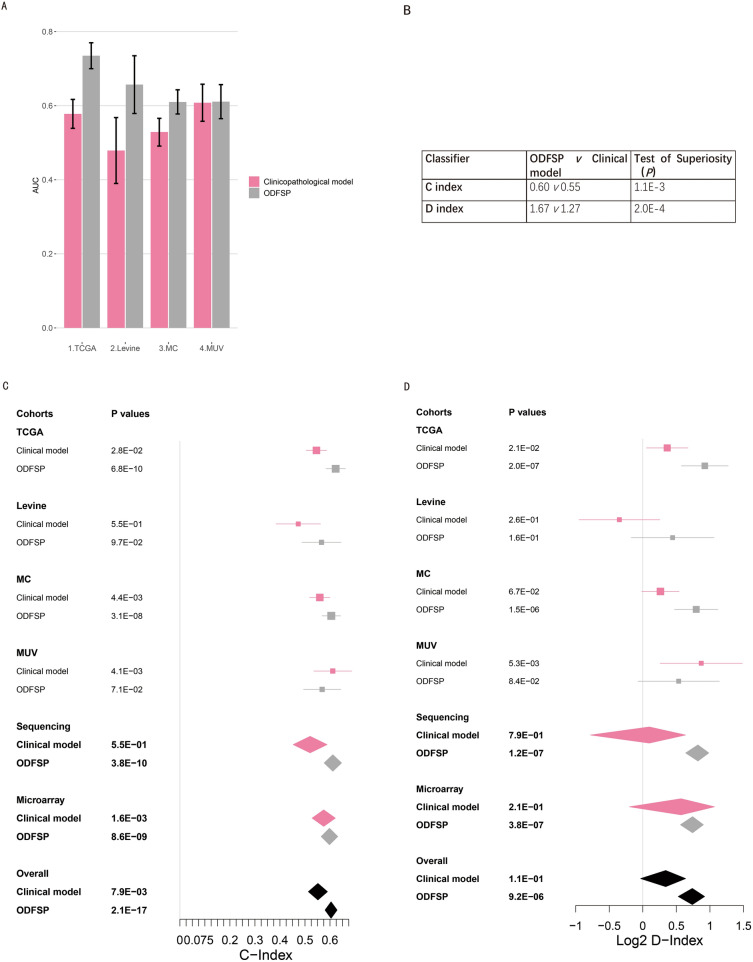
Comparison of the prognostic value of the clinicopathologic model and Ovarian Cancer Disease Free Survival Predictor (ODFSP). (**A**) Bar plot reporting the Area under the operating characteristics curve (AUC) for the clinical model and the ODFSP model. (**B**) The results of the test comparing the ODFSP and clinicopathological models for the meta C-index and meta D-index are shown in the tables. The Cancer Genome Atlas (TCGA), Levine cohort, Mayo Clinic cohort (MC), and Medical University of Vienna cohorts (MUV). (**C** and **D**) Forest plot reporting the (**C**) concordance index (C-index) and (**D**) D-index (robust hazard ratio) of validation cohorts computed using ODFSP and clinicopathologic model in the forest plot represent the point estimates, horizontal bars represent Confidence Interval (CIs), and the diamond is the meta-estimate.

The early recurrence of patients with OC was predicted using a logistic regression model fitted using these clinicopathologic variables. Given that in the univariable analysis (Data Supplement S4), age and tumor stage were significant and were used to create a clinicopathological model, the dataset was removed from this part because the NU cohort excluded age variable. The clinicopathologic model was insignificant in the sequencing cohort (D-index, 1.06, *P* = 0.79) or the array data sets (D-index, 1.48; *P* = 0.21, Fig. 5A). The clinicopathologic model's predictive value was compared to ODFSP in the Fig. 5B, C. ODFSP had a better predictive value than the clinicopathologic model (one-sided t test, *P* < 0.01; Fig. 5D).

### Prognostic models that have been published

**Figure 6:**
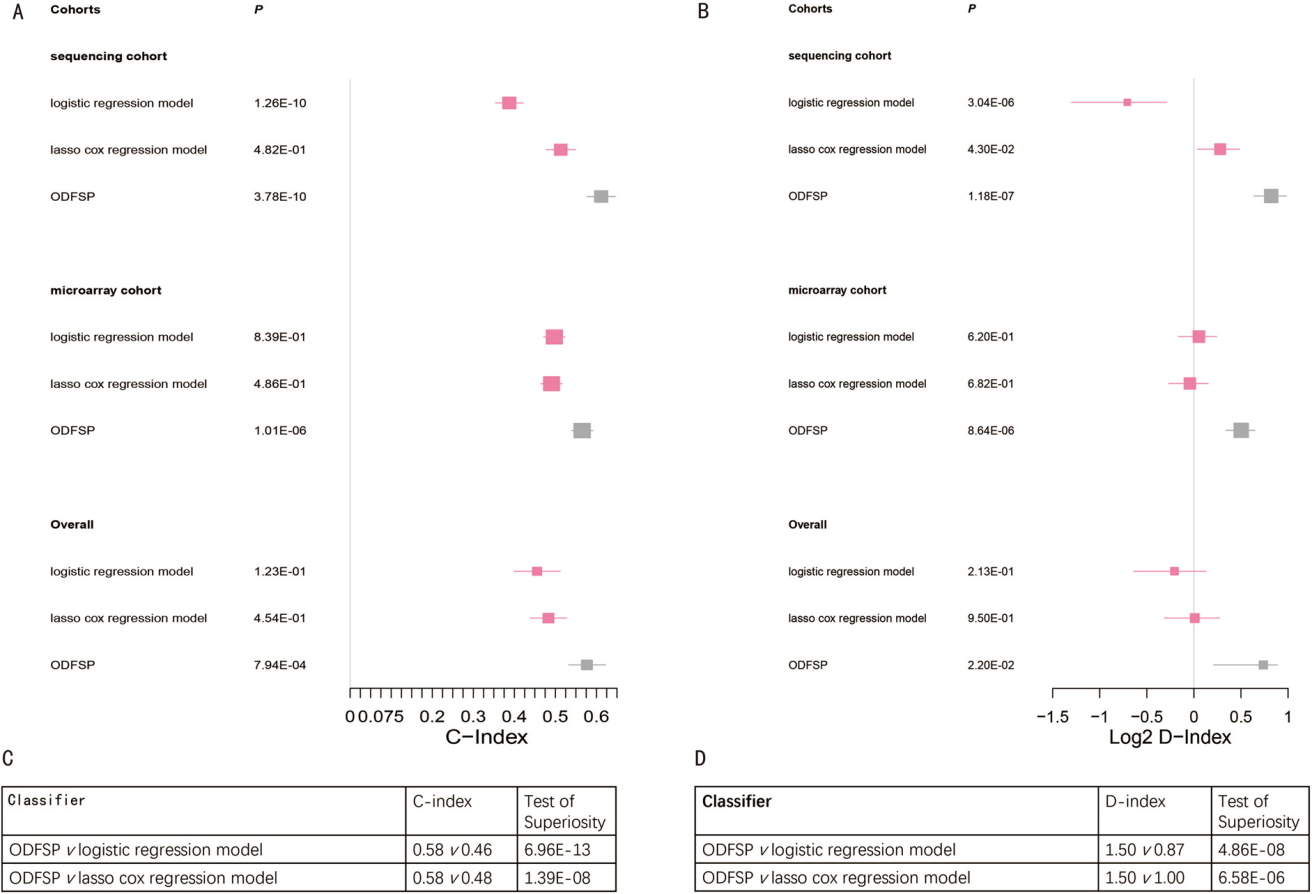
Comparison of existing classifiers with Ovarian Cancer Disease Free Survival Predictor (ODFSP). (A and B) Forest plot reports the meta-estimate of the (**A**) concordance index (C-index) and (**B**) D-index (robust hazard ratio) for ODFSP and existing classifiers. Squares in the forest plot represent the point estimates, horizontal bars represent CIs, and the diamond is the meta-estimate. (**C** and **D**) The table shows the result of test of superiority between ODFSP and different classifiers for (**C**) Meta C-index and (**D**) Meta D-index.

The prognostic value of ODFSP was compared to that of the logistic regression model and the lasso cox regression model, the latter of which was used in a study published in 2020^30^ to predict the overall survival of OC. ODFSP outscored them significantly in all cases (*P* < 0.05; Fig. 6A–D).

### Analysis of gene set enrichment for prognostic genes

**Figure 7:**
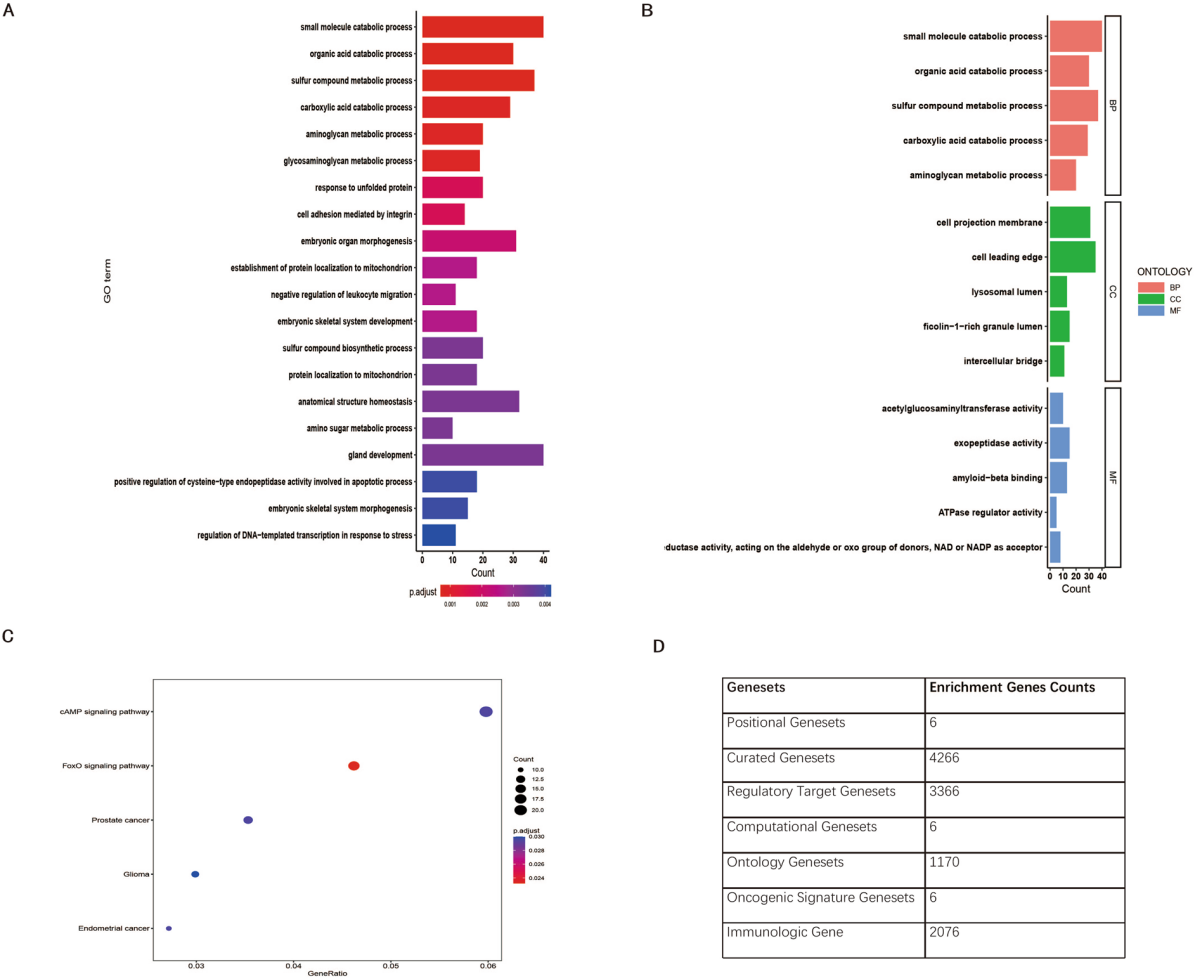
Analysis of Gene Set Enrichment for Prognostic Genes. (**A**) GO enrichment analysis of Ovarian Cancer Disease Free Survival Predictor (ODFSP) signature genes. The color intensity of the bars represents the number of enriched genes. (**B**) GO enrichment analysis of ODFSP signature genes divided into BP, CC and MF. (**C**) KEGG enrichment analysis of ODFSP signature genes. (**D**) Counts of Genes Enriched in Human MSigDB Collections.

Genes involved in small molecular catabolic process, sulfur compound metabolic process, organic acid catabolic process, sulfur compound biosynthetic process, glycosaminoglycan metabolic process, aminometabolic process, responded to unfolded protein were enriched in ODFSP at false discovery rate of less than 5% according to gene enrichment analysis for ODFSP signature genes (n = 886) (Fig. 7A, B, Data Supplement S5). KEGG analysis identified prominent pathways that included cAMP signaling pathway and FoxO signaling pathway (Fig. 7C). Figure 7D showed the counts of genes enriched in human MSigDB collections.

## Discussion

OC is a heterogeneous and genetically complex disease, and classifying its molecular biological and morphological characteristics can be a useful starting point for future therapeutic development. Based on a meta-analysis of 6 independent transcriptional datasets, we created ODFSP, a novel predictive model to highlight high-risk individuals for early recurrence. The meta-analysis employed the transcriptome profiles of 1580 OC patients, in which 1274 samples have recurrence information. The model was constructed using a unique tuple of 159 individuals who were evaluated using an array-based platform and a sequencing platform, and it was then confirmed using a summary of five different datasets containing 1115 patients. This meta-analysis framework maximizes model robustness and performance across cohorts for training and validating ODFSP. The ODFSP model has a high prognostic value for early recurrence (1 year or less) (*P* < 0.001).

Compared to other gene-related prognostic predictors, there is a lack of evidence supporting the efficacy of signatures initially designed for OC in predicting DFS, despite the abundance of prognostic signatures for overall survival (OS)^33,34^. In the context of DFS, ODFSP outperformed logistic regression and lasso cox regression models, both of which were trained on a small sample and never verified on larger datasets. In addition, when compared to other classifiers, the ODFSP can be utilized as a single sample predictor that is resistant to potential batch effects, profiling platforms, and normalization approaches. ODFSP model avoids batch impact by simplifying continuous expression space into binary pair barcodes. Based on the same reason, ODFSP also outperforms prevailing models in both microarray and sequencing platforms. In addition, the ODFSP model demonstrated cross-platform stability to various normalization methods.

In contrast to known predictive clinicopathologic markers, the ODFSP model predicted recurrence better than the clinicopathologic model. The ODFSP model had considerable predictive significance even after correcting for clinicopathologic factors (age, FIGO stage).

The ODFSP model contains 886 unique genes and 2040 unique gene pairs. Genes involved in small molecular catabolic process, sulfur compound metabolic process, organic acid catabolic process, sulfur compound biosynthetic process, glycosaminoglycan metabolic process and aminometabolic process, was found to be enriched in a functional study of 886 genes. For KEGG analysis of ODFSP signature genes, cAMP signaling pathway and FoxO signaling pathway were identified, which were suggested in related with cAMP signaling pathway and FoxO signaling pathway in ovarian neoplasms^35^.

The 886-gene ODFSP model should be reduced for wider utilization in clinical setting. Therefore, we examined alternative feature set sizes for the k-Top scoring disjoint pairs models and assessed the performance of the reduced models. In AUROC, we achieved equivalent performance to the 886-gene ODFSP model by integrating only 177 unique genes (Data Supplement S5), indicating that smaller ODFSP-like models could be used in clinical situations (Appendix A2). The reduced ODFSP can be used to evaluate the prognosis of patients with OC before undergoing curative surgery, assisting doctors in selecting patients whom surgery is optimal and identifying high-risk progressing cases wherein surgery proves minimally beneficial^36^.

The existing research had some fragilities. Foremost is the incorporation of bias-accumulating intrinsic tumor sample that were obtained from a variety of data sets, sampled at varying locations, and different hospitals having unparalleled standard of care. The MC cohort and NU data sets were macro dissected, while the TCGA/ICGC data sets profiled bulk tumors. Second, generation of transcriptomic profiles in our data compilation was achieved by using distinct microarray platforms (Agilent Technologies, Santa Clara, CA; Affymetrix, Santa Clara, CA; and Illumina) and distinct gene expression profiling methods were adopted for sequencing (Illumina HiSEquation 2000/2500; Illumina, San Diego, CA). Third, all samples were normalized using publicly available processing methods that are dependent on the profiling platforms (Data Supplement S1). Furthermore, transforming expression data into binary barcodes may result in information loss in terms of co-expression and amount of differential expression between genes. However, the binary barcodes strategy has statistical advantages over predictions depending on continuous gene expression data. The binary barcode method generates single-sample predictions that are unaffected by monotonic transformations of gene expression data, which is especially useful in meta-analyses of heterogeneous cohorts where continuous gene expression–based prediction approaches require data scaling for comparison across cohorts.

Despite these flaws, ODFSP produced consistent predictive value across varied data sets, implying that the gene expression barcode transformation is resistant to the unavoidable biases that plague massive meta-analyses. To improve the prediction accuracy of predictive models, more research into germline variants, epigenetics, copy number alterations, noncoding RNAs, protein abundance, and epidemiologic and environmental factors will be required. Our meta-analysis is further limited by the absence of available clinical treatment information, especially treatment, across cohorts, deterring us from delving into this source of heterogeneity. However, when comparing cohort-specific clinical information, there were no significant differences between cohorts (Data Supplement S2). The standard-of-care treatment for ODFSP at the time of sample collection was curative-intent surgery accompanied by adjuvant chemotherapy with platinum and taxane. Maintenance with bevacizumab, the antibody–drug combination mirvetuximab soravtansine co-administered with bevacizumab, has shown anti-tumor activity that leads to long-term responses in platinum “agnostic” (resistant/sensitive) casesy^37^. Many sites are also evaluating the effect of neoadjuvant chemotherapy and antibody–drug conjugates; consequently, therapeutic variability is expected within and between cohorts. It may be essential to test the ODFSP model with additional clinical cohorts or, ideally, randomized trials.

Finally, we employed the maximum possible set of OC transcriptomes to create ODFSP, a predictive model that detects OC-diagnosed participants who are at high risk of early recurrence and outperforms such models established on clinicopathologic characteristics or molecular subtypes. ODFSP could be used in the clinic as a single sample classifier to identify patients who are at a higher risk of relapsing early following surgery and adjuvant chemotherapy, thereby easing treatment decisions.

### Supplementary Information

Supplementary Information 1.

Supplementary Information 2.

Supplementary Information 3.

Supplementary Information 4.

## Data Availability

The datasets generated and analysed during the current study are publicly available at https://dcc.icgc.org, https://portal.gdc.cancer.gov/projects/TCGA-OV, https://www.ncbi.nlm.nih.gov/geo/query/acc.cgi?acc=GSE102094, https://www.ncbi.nlm.nih.gov/geo/query/acc.cgi?acc=GSE140082, https://www.ncbi.nlm.nih.gov/geo/query/acc.cgi?acc=GSE32062, and https://www.ncbi.nlm.nih.gov/geo/query/acc.cgi?acc=GSE49997. Our code is publicly available on https://github.com/wanlei1618/ODFSP.
