# Supplementary material for: A prognostic model for ovarian neoplasms established by an integrated analysis of 1580 transcriptomic profiles
Source: Sci Rep. 2023 Nov 8;13:19429. doi: 10.1038/s41598-023-45410-x (PMC10632395; doi:10.1038/s41598-023-45410-x)
Supplement: Supplementary file 1 — Supplementary Information 1. [file 41598_2023_45410_MOESM1_ESM.docx]

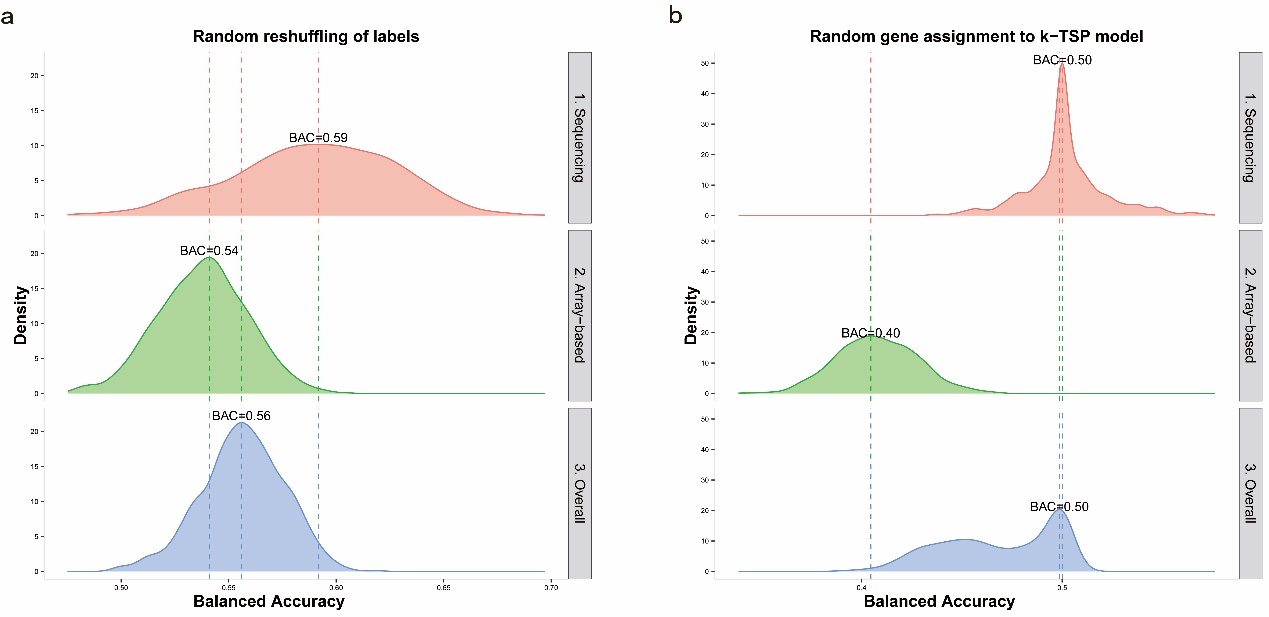


Appendix Figure A1. Density plots are presented to illustrate the distribution of balanced accuracy (BAC) for random models. In panels (a) and (b), the distribution of meta-estimates is shown for 1,000 models generated through (a) random reshuffling of labels and (b) random assignment of genes to k-Top scoring disjoint pair (k-TSP) models. The meta-estimates were calculated independently for the combined cohorts, sequencing cohorts, and array-based cohorts. The pink, green, and blue dashed lines correspond to the meta-estimates of the area under the receiver operating characteristic curve obtained from the Ovarian Cancer Disease Free Survival Predictor (ODFSP) for the overall, sequencing, and array-based cohorts, respectively.


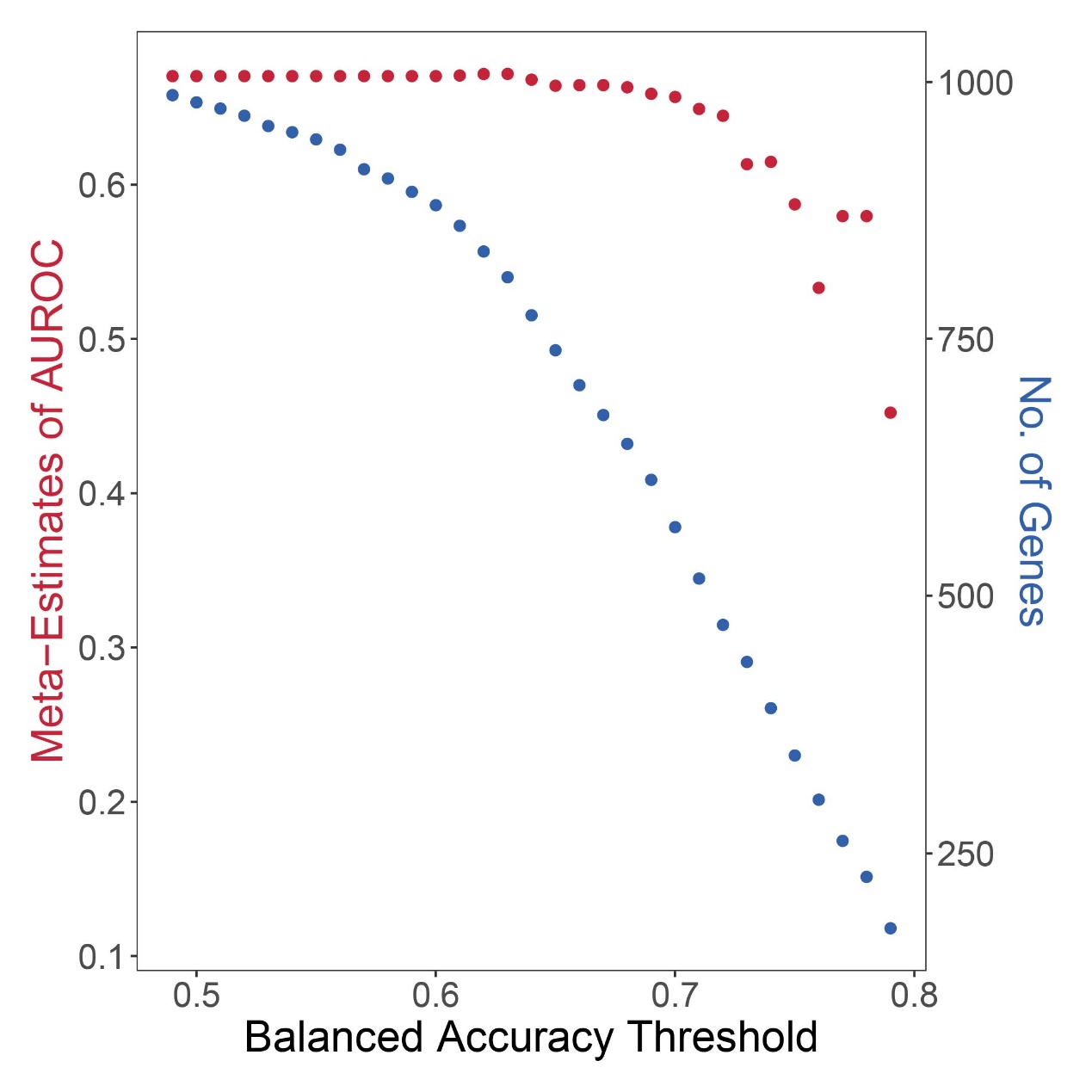


Appendix Figure A2. The scatterplot depicts the correlation between the meta-estimate of the area under the receiver operating characteristics curve (AUROC), denoted by the color red, and the total number of unique genes, denoted by the color blue, in the Ovarian Cancer Disease Free Survival Predictor (ODFSP) model at various balanced accuracy thresholds.
