# Supplementary material for: A prognostic model for ovarian neoplasms established by an integrated analysis of 1580 transcriptomic profiles
Source: Sci Rep. 2023 Nov 8;13:19429. doi: 10.1038/s41598-023-45410-x (PMC10632395; doi:10.1038/s41598-023-45410-x)
Supplement: Supplementary file 2 — Supplementary Information 2. [file 41598_2023_45410_MOESM2_ESM.docx]

| **Datasets** | **Platform** | **No. of samples** | **With recurrence data** | **Normalization method** | **Reference** |
| --- | --- | --- | --- | --- | --- |
| ICGC(sequencing & microarray) | Illumina HiSeq/Affymetrix U133Aa | 262(sequencing 373; microarray 567) | 159 | Lowess |  |
| TCGA | Illumina GAIIx | 379 | 243 | RSEM | Nature. 2011;474(7353):609-615. doi:10.1038/nature10166 |
| Levine | Illumina HiSeq 2500 | 95 | 80 | TMM | Nat Commun. 2017; 8(1):990. doi: 10.1038/s41467-017-01217-9. |
| MC | Ilumina HumanHT 12 V4 probe | 380(Serous:277） | 362(265） | Log quantile normalization | Clin Cancer Res. 2017; 23(14): 3794–3801. doi: 10.1158/1078-0432.CCR-16-2196 |
| NU | Agilent-014850 Whole Human Genome Microarray 4x44K G4112F | 260 | 258 | TMM | Clin Cancer Res. 2012; 18(5):1374-85. doi: 10.1158/1078-0432.CCR-11-2725 |
| MUV | ABI Human Genome Survey Microarray Version 2 | 204(Serous:171) | 172(151） | quantile normalization | Cancer Sci. 2012; 103(7): 1334–1341. doi: 10.1111/j.1349-7006.2012.02306.x |

**Data Supplement S1: The table shows the datasets used in the project for meta-analysis**
