# Supplementary material for: A prognostic model for ovarian neoplasms established by an integrated analysis of 1580 transcriptomic profiles
Source: Sci Rep. 2023 Nov 8;13:19429. doi: 10.1038/s41598-023-45410-x (PMC10632395; doi:10.1038/s41598-023-45410-x)
Supplement: Supplementary file 3 — Supplementary Information 3. [file 41598_2023_45410_MOESM3_ESM.docx]

**Data Supplement S2: The table shows the clinicopathological information of the cohorts used in the analysis.**

| Cohorts |  | ICGC-micro/seq | TCGA | Levine | MC | NU | MUV |
| --- | --- | --- | --- | --- | --- | --- | --- |
| Figo | I | 0 | 1 | 1 | 19 | 0 | 0 |
|  | II | 11 | 12 | 3 | 29 | 0 | 9 |
|  | III | 128 | 194 | 52 | 255 | 202 | 139 |
|  | IV | 20 | 36 | 24 | 59 | 56 | 24 |
| Grade | G1 | 0 | 0 |  |  | 0 | 43（G1/G2) |
|  | G2 | 21 | 30 |  |  | 129 | 43（G1/G2) |
|  | G3 | 133 | 206 |  |  | 129 | 128 |
|  | G4 | 1 | 1 |  |  | 0 | 0 |
|  | GB | 1 | 1 |  |  | 0 | 0 |
|  | GX | 2 | 4 |  |  | 0 | 0 |
|  | Null | 0 | 1 | 0 | 22 | 0 | 1 |
|  | high grade |  |  | 80 | 270 |  |  |
|  | low grade |  |  | 0 | 70 |  |  |
| Median DFS |  | 1.26 | 1.23 | 1.75 | 1.55 | 1.58 | 1.42 |
| Median Age |  | 56.76 | 58.5 | 59 | 59 | Not available | 58 |
| Adjuvant Rx | Pre-Adjuvant Therapy | 19 | 43 | Not available |  | Not available | Not available |
|  | Post-Adjuvant Therapy | 16 | 20 | Not available |  | Not available | Not available |
|  | Preoperative Therapy | 7 | 16 | Not available |  | Not available | Not available |
|  | Other | 12 | 11 | Not available |  | Not available | Not available |
|  | bevacizumab | Not available | Not available | Not available | 194 | Not available | Not available |
|  | Null | 105 | 153 | Not available |  | Not available | Not available |
| histology | serous | 159 | 243 | 80 | 265 | 258 | 151 |
|  | Other | 0 | 0 | 0 | 97 | 0 | 21 |
